# Ethics dumping in artificial intelligence

**DOI:** 10.3389/frai.2024.1426761

**Published:** 2024-11-08

**Authors:** Jean-Christophe Bélisle-Pipon, Gavin Victor

**Affiliations:** ^1^Faculty of Health Sciences, Simon Fraser University, Burnaby, BC, Canada; ^2^Philosophy Department, Simon Fraser University, Burnaby, BC, Canada

**Keywords:** artificial intelligence, AI ethics, ethics dumping, ethical guidelines, accountability, AI governance

## Abstract

Artificial Intelligence (AI) systems encode not just statistical models and complex algorithms designed to process and analyze data, but also significant normative baggage. This ethical dimension, derived from the underlying code and training data, shapes the recommendations given, behaviors exhibited, and perceptions had by AI. These factors influence how AI is regulated, used, misused, and impacts end-users. The multifaceted nature of AI’s influence has sparked extensive discussions across disciplines like Science and Technology Studies (STS), Ethical, Legal and Social Implications (ELSI) studies, public policy analysis, and responsible innovation—underscoring the need to examine AI’s ethical ramifications. While the initial wave of AI ethics focused on articulating principles and guidelines, recent scholarship increasingly emphasizes the practical implementation of ethical principles, regulatory oversight, and mitigating unforeseen negative consequences. Drawing from the concept of “ethics dumping” in research ethics, this paper argues that practices surrounding AI development and deployment can, unduly and in a very concerning way, offload ethical responsibilities from developers and regulators to ill-equipped users and host environments. Four key trends illustrating such ethics dumping are identified: (1) AI developers embedding ethics through coded value assumptions, (2) AI ethics guidelines promoting broad or unactionable principles disconnected from local contexts, (3) institutions implementing AI systems without evaluating ethical implications, and (4) decision-makers enacting ethical governance frameworks disconnected from practice. Mitigating AI ethics dumping requires empowering users, fostering stakeholder engagement in norm-setting, harmonizing ethical guidelines while allowing flexibility for local variation, and establishing clear accountability mechanisms across the AI ecosystem.

## Introduction

It is widely accepted that technologies are not value-neutral. This is especially true for complex and impactful technologies like artificial intelligence (AI) (Elliott, 2019; Alami et al., 2020). Beyond encoding statistical models and procedural rules, AI systems embody significant ethical baggage derived from their underlying code and the data used (Norori et al., 2021). This normative dimension shapes AI systems’ recommendations, behaviors, and decision-making processes. It also influences how AI is perceived, regulated, used (or misused), and how it impacts different stakeholders, especially end-users (Berberich et al., 2020). AI’s undeniable power has sparked discussions in domains such as ELSI (ethical, legal, and social implications), STS (Science and Technology Studies), and applied ethics (Dubber et al., 2020; Hagendorff, 2020; Horgan et al., 2020; Bélisle-Pipon et al., 2021; Slota et al., 2021). A recurring theme is that technologies can impose values and norms exogenous to the contexts where they are deployed (e.g., financial technologies marginalizing those without banking access, precision agriculture conflicting with traditional practices and biodiversity, advanced medical equipment ill-suited for local healthcare infrastructures, and surveillance systems raising privacy concerns in diverse social contexts and norms). Such value imposition necessitates critical analysis of an AI’s ethical underpinnings. In the wake of rising concerns over AI’s societal impacts, a flourishing ecosystem of scholarship and initiatives has emerged to develop ethical frameworks and guidelines for responsible AI development and use (Hagendorff, 2020). However, this initial wave prioritized articulating high-level principles over examining practical challenges in norm design and implementation (Lauer, 2020). The field advanced cautiously, outlining a conceptual map of AI ethics imperatives which had previously been uncharted.

As the discourse matures, increasing attention is devoted to the challenges of translating ethical principles into practice and developing robust governance mechanisms (Mittelstadt, 2019; Ryan and Stahl, 2020). Key concerns include responsibly embedding ethics into design and deployment pipelines (Floridi et al., 2020), evaluating normative impacts across contexts (Eitel-Porter, 2020), and establishing accountability structures (Russell et al., 2015). Something not often considered, though, is that ethical guidelines and accountability expectations can be unfairly placed on users and impacted communities, leading to an unfair normative burden if developers and regulators offload responsibility without empowering local agency. To further investigate this dynamic, we look to the concept of “ethics dumping” in research ethics, which highlights how the export of ethical guidelines and governance from powerful actors to less-privileged contexts can impose disproportionate costs and burdens if local conditions are not accounted for (Schroeder et al. 2019a). Drawing on this framing, the present paper argues that practices surrounding the development, deployment and governance of AI can lead to different forms of ethics dumping, thereby unduly burdening, and even harming, users and host environments who may lack the capacities or agency to responsibly manage the embedded normative content and ethical implications.

The paper begins by outlining the ethics dumping concept and its significance within research ethics. It then examines four key trends illustrating how different AI actors—developers, principles/guideline authors, institutional implementors, and regulators/policymakers—can each contribute to ethics dumping through their practices. Finally, potential mitigation strategies are discussed, emphasizing approaches that empower users/host environments, foster stakeholder inclusion, harmonize ethical guidelines while allowing flexibility for local variation, and establish clear accountability structures across the AI ecosystem.

## Ethics dumping: a borrowed concept

In its simplest conception, ethics dumping refers to the export of ethically dubious practices from a privileged context to one with weaker governance mechanisms (Schroeder et al., 2016). Originating in research ethics, the concept highlights how problematic behaviors occur more frequently when studies are conducted in resource-poor settings “with weaker compliance structures or legal governance” (Schroeder et al., 2019b). Classic examples include drug trials that exploit regulatory gaps or institutional deficiencies in developing countries, undermining robust ethical protocols, and imposing disproportionate risks on local populations who may not share in the eventual benefits. Beyond physical and economic asymmetries, the ethics dumping concept also captures how ethical principles and accountability norms flow from powerful actors to recipient communities without their participation or ability to shape those norms. At its core, ethics dumping emerges from skewed power dynamics, where certain downstream populations or environments are unable to negotiate or uphold ethical practices aligned with their values and contexts (Schroeder et al., 2019b; Samuel and Derrick, 2020).

The European Union (EU)-funded TRUST project developed guidance for addressing and counteracting ethics dumping in international research partnerships (Andanda et al., 2014). They trace the concept of ethics dumping to a more general phenomenon, “dumping.” In the global economic and trade sectors, dumping is a form of predatory pricing. Dumping refers to cases where “large entities can afford to undercut local competitors for a given period, to drive them out of the market” (Andanda et al., 2014). By doing so, the companies “dump” their product into a new environment in a manner that harms said environment by predating on local competition. Viewed as a form of dumping, though ethics dumping tends to be described in terms of international research practices, it may also be understood as a more general predatory phenomenon: placing ethical responsibility in a domain which lacks the ability to incorporate that responsibility into its operations.

In the context of biomedical research, the concept of ethics dumping is further elaborated by Liao et al. (2023) in their analysis of its occurrence within China. They define ethics dumping as the practice where researchers, often from countries with stringent ethical regulations, conduct ethically questionable research in regions with less rigorous oversight, exploiting local vulnerabilities. The authors highlight how China, with its relatively weaker ethical governance structures, becomes a target for such practices. They detail cases like the CRISPR baby scandal and the Golden Rice incident to illustrate how ethics dumping manifests in biomedical research, where the pursuit of scientific advancement or personal gain overrides ethical considerations, often at the expense of local populations. The authors emphasize that ethics dumping is not merely a transfer of unethical practices but often a deliberate circumvention of ethical norms, facilitated by gaps in local oversight and regulation, leading to significant ethical breaches and harm. However, they acknowledge that it may sometimes occur unintentionally, as individuals operating in unfamiliar contexts might unknowingly engage in inappropriate practices due to insufficient understanding or awareness of relevant ethical considerations (Liao et al., 2023).

The ethics dumping concept provides a useful lens for examining dynamics within AI ethics. Although the AI context differs from research ethics, similar power asymmetries and risks of offloading normative content exist between developers of increasingly autonomous and opaque systems, and end-users expected to responsibly deploy those systems. The following section outlines four key trends illustrating how practices from different actors across the AI ecosystem can contribute to ethics dumping.

## AI ethics dumping: four concerning trends

Like other technologies where ethical implications cut across domains, defining responsible practices for AI has catalyzed a “race” by numerous organizations aiming to shape the normative discourse and establish ethical guardrails (Whittlestone et al., 2019; Keller and Drake, 2020). However, despite this flurry of activity articulating principles and guidelines, concerns remain over how well AI ethics translates into practice (Ho, 2020). Local norms, constraints, and stakeholder values risk being overridden by one-size-fits-all frameworks (Chaudhary, 2020). More insidiously, skewed power dynamics mean that those developing ethical guidelines or deploying AI systems may effectively dictate normative standards that impacted communities must then bear primary responsibility for upholding, even if poorly equipped or excluded from shaping those standards. This section identifies four key trends (see Table 1) that can enable ethics dumping in the AI context, highlighting how different actors—developers, ethics principle-authors, institutional implementors, and regulators—may each facilitate the unfair transfer of ethical burdens and responsibilities.

**Table 1:** Summary of ethics dumping trends in artificial intelligence.

Trend	Description	Key Features
Trend 1: ethics dumping by AI developers	AI developers exert disproportionate influence over ethical norms through design choices and embedded values in algorithms. This can result in users unknowingly adopting biased or misaligned norms due to the opaque nature of AI systems.	Norms encoded in AI design Lack of transparency Users adopt technology without the means to implement ethical safeguards
Trend 2: ethics dumping by ethics guideline authors	Broad and vague ethical guidelines, developed without local stakeholder engagement, place the burden on companies/developers or end-users to interpret and apply these principles within their specific contexts, leading to ethics dumping.	Vague principles difficult to translate into actionable items Disconnect from operational realities Users bear responsibility for implementation
Trend 3: ethics dumping by institutional implementors	Institutions adopting AI systems often prioritize deployment over ethical evaluation, resulting in a shift of responsibility for ethical and legal compliance to end-users, who may lack the resources to manage these responsibilities.	Context of rapid AI deployment Inadequate ethical evaluation Responsibility shifted to end-users
Trend 4: ethics dumping via governance frameworks	Governance for large-scale AI solutions often lack inclusive processes, imposing norms without considering local contexts, and shifting compliance burdens onto users.	Top-down governance Lack of thorough stakeholder inclusion One-size-fits-all ethical norms Shifted compliance responsibilities to users

### Trend 1: ethics dumping by AI developers

Like all technologies, AI systems have profound impacts on users and their environments that go beyond just technical functionality (Alami et al., 2020; ÓhÉigeartaigh et al., 2020). What fuels AI’s transformative promise and “hype” is precisely its potential to significantly reshape practices, beliefs, and decision-making processes (Matheny et al., 2019; Char et al., 2020). However, this transformative capacity means users are not just adopting a tool, but also the normative priors embedded within it. As highly opaque and autonomous systems, developers often have disproportionate influence in shaping AI’s ethical dimension by encoding inferences, assumptions, and values into algorithms and training datasets (Rahwan, 2018; ; McDermid et al., 2021). AI does not neutrally reflect the world but actively frames and constructs it through the choices and constraints inherent in its design (Barocas and Selbst, 2016; Mittelstadt et al., 2016). Datasets skewed by historical biases and human developer assumptions about relevant features mean AI outputs can reflect and perpetuate specific cultural viewpoints, hierarchies, and injustices (Buolamwini and Gebru, 2018). While typically undertaken with no malicious intent, allowing values to creep into algorithms in this manner facilitates ethics dumping in several ways. First, by implicitly encoding norms within AI systems’ architecture (designed in a specific cultural/institutional milieu), users adopt this “black boxed” normative baggage upon deployment even if antithetical to local values or accountability customs (Ananny and Crawford, 2018; Crawford, 2021; Bélisle-Pipon et al., 2021). That is, users might be interacting with an algorithm that embodies values which do not align with their own, but AI opacity prevents them from recognizing whether this is even the case. Second, despite the vast influence of developer ethics-by-design choices (such as enhancing privacy protection and data security, mitigating bias, and seeking to improve fairness in algorithms), the opacity and inscrutability of most AI systems leave users with little insight into the rationale or assumptions driving system behaviors, and leave them with little means of addressing the normative considerations baked into the algorithms (Burrell, 2016; Akhai, 2023; Stafie et al., 2023). Finally, the very distance between developers and where impacts manifest means developers are not the ones who will have to manage the impacts of their technology (Crawford and Calo, 2016). This misalignment between influence over normative design choices and accountability for downstream effects enables ethics dumping—it allows AI use contrary to exemplary or even local standards if communities lack resources to investigate and intervene.

As an example, consider OpenAI’s recent controversy surrounding watermarking ChatGPT text outputs. Though the company has been developing a watermarking feature for about a year, it has decided against implementing the feature for fears that it will result in less usage (Davis, 2024). By not instituting a watermark, OpenAI places responsibility on downstream stakeholders to find mechanisms of accountability for users. Importantly, OpenAI bases its decision not on increasing calls for responsible usage of large language models, but with surveyed users, almost 30% of whom would be less likely to use ChatGPT with a watermark (Davis, 2024). The company therefore has the means to act, but faced with a strategic loss, has decided not to make the use of its product more transparent and traceable. In doing so, ChatGPT is “dumped” into the market—a market which is not clearly prepared to deal with the ramifications of the product—while also entrusting these users with responsibilities for accountability attribution, even if it is far more difficult for those outside OpenAI to institute accountability structures.

Some suggest that ethics-by-design can resolve these tensions by hardwiring ethical guidelines into algorithms from inception (Dignum, 2018). However, self-regulation is an insufficient solution if the processes around defining and embedding ethical norms lack meaningful stakeholder participation and oversight. Without inclusive and thorough norm-setting structures, ethics-by-design risks amplifying ethics dumping by concentrating normative influence in the hands of developers and institutions least accountable to impacted populations (Hasselbalch, 2021; Fraenkel, 2024; Umbrello, 2024).

### Trend 2: ethics dumping by AI ethics guideline authors

The recent profusion of AI ethics guidelines and principles represents a shared aspiration to normatively constrain the development and application of transformative AI systems. However, their very generality and lack of context-specific stakeholder engagement, counterintuitively, can *enable* ethics dumping. Most ethical AI principles articulate high-level injunctions like transparency, fairness, accountability, and respect for human rights that few would disagree with (Jobin et al., 2019). Well-intentioned as ethical principles are, their vagueness and distance from operational realities risk creating an “ethics buffer” (or ethics washing) that signals virtuous intent while doing little to guide on-the-ground implementation by those bearing responsibility for upholding such principles (Vakkuri et al., 2019). This “ethics buffer” dynamic engenders ethics dumping because broad principles developed without local stakeholder buy-in place disproportionate burdens on users (Criado-Perez, 2019).

Consider, for example, the White House “Blueprint for an AI Bill of Rights” and its call for notices informing those impacted by AI systems (The White House, 2022). The Blueprint states that

Designers, developers, and deployers of automated systems should provide generally accessible plain language documentation including clear descriptions of the overall system functioning and the role automation plays, notice that such systems are in use, the individual or organization responsible for the system, and explanations of outcomes that are clear, timely, and accessible (The White House, 2022).

The Blueprint does well to specify what is meant by a notice, so that the notice is clear, informative, and relevant. Important as disclosures and notices may be, this call transfers ethical responsibility to “designers, developers, and deployers” (The White House, 2022). While this may seem normal when it comes to such high-level guidance, the problem is this: the guidelines do not seem to specify *when developers* are under the obligation to provide a notice versus when the *deployers* are under the obligation to provide a notice. This leaves a vacuum of guidance, and the expectation that those involved in the creation and rollout of the algorithm will fill this vacuum. But, designers, developers, and deployers do not have ethical standards by which they can arbitrate between each other’s role in the process of forming a notice. In the absence of more clear responsibilities held by parties involved, guidance like the Blueprint takes a step in the right direction, but also passes ethical responsibilities to parties lacking the structures by which to embody and carry out these responsibilities.

Responsibility and accountability flow towards practitioners although they lacked agency and authority in defining the principles expected of them. Care is a central aspect of responsibility, especially toward people in vulnerable situations, such as those in resource-poor areas. When those tasked with supporting these individuals fail to provide adequate care, there is a risk of exploitation or “dumping.” This can occur when limited capacity and expertise hinder effective governance, leading to insufficient protections for those in need (Schroeder et al., 2019b). Some AI ethics guidelines do acknowledge the importance of stakeholder engagement and responsiveness to local norms (Fjeld et al., 2020). However, most guideline development processes have been criticized for marginalizing or excluding key stakeholders like minority groups and civil society (Bélisle-Pipon et al., 2022). Without inclusive representation at a principle’s conception, enforcing guidelines across diverse cultural contexts enables ethics dumping by disconnecting normative ideals from local realities. Importantly, this dumping dynamic extends beyond guidelines produced by industry or multi-stakeholder bodies. Well-intentioned guidance from respected institutions, if developed through insular processes insensitive to on-the-ground complexities, paradoxically facilitates dumping by advocating one-size-fits-all accountability checklists that practitioners must then struggle to reinterpret and implement (Rakova et al., 2021; Khosravi et al., 2022; Bennett et al., 2023). Ethics dumping here entails burdening frontline actors with operationalizing academic philosophies abstracted away from pragmatic realities and without accounting for contextual variation.

### Trend 3: ethics dumping by institutional AI implementors

A third manifestation of AI ethics dumping emerges from the practices of institutions acquiring and deploying AI systems. Well-intentioned efforts to leverage transformative AI capabilities can prompt adoption of systems whose embedded normative logics and accountability implications were not robustly evaluated from an ethical lens (Taddeo and Floridi, 2018).

For example, in healthcare settings, initiatives to deploy AI predictive and diagnostic tools, clinical decision support systems (CDSS), AI-generated in-basket responses, or patient monitoring tools tout promises of increased efficiency and optimized resource allocation (Chen et al., 2021; Elhaddad and Hamam, 2024; Garcia et al., 2024; Khosravi et al., 2024). However, ethical issues like eroding human decision autonomy, entrenching biases from historical data, and disrupting professional role boundaries often remain underexplored during acquisition and piloting phases (Char et al., 2018). Pressing user needs and institutional enthusiasm catalyze a “push” dynamic where normative downsides are discounted in the rush towards AI deployment (Lindgren, 2023; Gray and Shellshear 2024). Medical institutions’ policies can negatively impact caregivers, administrative staffs, patients, relatives, and stakeholders by shifting the responsibility to them to supervise the use of institution-approved or promoted tools. This often entails failing to provide necessary frameworks, usage agreements, best practices, or terms of reference to guide the implementation, evaluation, and eventual decommissioning of medical AI tools. If the institution does not take into account the complete lifecycle of a medical AI, and does not act as appropriate gatekeepers, this will have dumping impacts on downstream uses within the institution, and carry not only health, but also ethical and legal risks.

Even when ethical risks and implications are assessed, implementors’ incentives frequently align more with signaling ethical conduct through procedural checklists rather than committing resources to substantive mitigations (Raji et al., 2020). As with ethics guidelines, the push dynamic stems from implementor institutions lacking robust governance structures ensuring inclusive participation of stakeholders impacted by new AI deployments. When affected communities are not involved in co-defining metrics, risk thresholds, and ethical guidelines, institutions deploying AI systems may inadvertently engage in practices resembling ethics dumping. Such approach often centralizes control over data systems among institutional actors, while limiting transparency, accountability, and public engagement, thereby leaving communities with little agency over impactful decisions (Dencik et al., 2018). This asymmetry between normative authority and experiential burden reflects a central dynamic underlying ethics dumping.

### Trend 4: ethics dumping via governance frameworks

Many argue that to ensure responsible and trustworthy AI, ethical norms need to be considered from the outset (and not be an afterthought) of the development and deployment of an AI solution, as well as adopting an inclusive, open and transparent approach, especially when it affects a very large number of people or is a public/governmental solution (Dobbe et al., 2021; Couture et al.,2023). This is particularly crucial for the responsible development and deployment of large-scale AI solutions aimed at population-wide or population-specific impacts. Centralized legal/regulatory governance frameworks have advantages of uniformity, reducing ethical hazards from unconstrained corporate self-interest, and institutionalizing public accountability mechanisms. However, many governance frameworks for large-scale AI solutions present a concerning lack of sustained stakeholder inclusion and bottom-up norm-setting, at the risk of neither considering nor responding to the real needs of end-users, but above all, in our case, of making them bear the burden of accountability for the implementation and responsible, trustworthy use of said AI solution. Their development is too frequently steered by regulators, legislators, and entrenched dominant (often coming from industry) incumbents leveraging their advantageous position in the AI sector to guide the development of governance (Bélisle-Pipon et al., 2022). Even well-intentioned civil society voices often find their perspectives marginalized in the closed-door negotiations that shape these rules.

The AuroraAI program in Finland serves as a notable example of how AI ethics dumping can manifest through inadequate governance. AuroraAI was an ambitious initiative aimed at integrating AI into public service delivery to enhance efficiency and provide personalized, data-driven support to citizens (Finnish Center for Artificial Intelligence, 2023). This was set to be the first independent AI assistant dedicated to public services (ODSC-Open Data Science, 2018). While the program’s goal of improving individual well-being through seamless, AI-facilitated interactions is laudable, the ethical oversight of the project has been inadequate. The governance model for AuroraAI has been largely top-down, with critical decisions and ethical considerations being handled by a central Ethics Board established only after significant progress had already been made on the project and which had limited influence over the pre-existing goals and decisions (Leikas et al., 2022).

This delayed and centralized approach to ethical oversight is emblematic of AI ethics dumping, where the burden of managing ethical implications is shifted away from the developers and policymakers who designed the system onto local users and communities who interact with it daily. The limited public engagement in the ethical deliberation process, coupled with the program’s broad and generalized ethical guidelines, has resulted in a scenario where the responsibility for addressing complex ethical challenges is disproportionately placed on those least equipped to manage them (Algorithm Watch, 2020). Some raised concerns about “ethics washing” (Leikas et al., 2022). This dynamic not only sidelines broader societal input but also risks embedding ethical standards that may not align with local values or contexts. As a result, the AuroraAI program, despite its human-centric objectives, exemplifies how a governance framework can facilitate ethics dumping by imposing a one-size-fits-all model that overlooks the need for localized ethical consideration and sustained stakeholder engagement.

When governance frameworks for large-scale AI solutions are developed without inclusive processes and are disconnected from the specific realities of end-users, they can exacerbate AI ethics dumping at least three ways. First, by encoding ethical principles favored by developers and policymakers insensitive to minority group needs, governance creates downstream burdens for users to uphold norms shaped without their input (Tallberg et al., 2023). Second, prescriptive top-down design and ruling force a “one-size-fits-all” paradigm on diverse contexts, overriding local values and accountability customs (Cinnamon, 2019). Finally, such governance frameworks frequently concentrate liability risk and regulatory compliance responsibilities on frontline users rather than on developers and institutional procurers of AI systems (Duffourc et al., 2023). Even robust public engagement processes often suffer from tokenism, with participatory mechanisms ill-equipped to translate marginal voices into pragmatic reforms. Consequently, despite progress in AI ethics since revelations of bias, discrimination, and lack of accountability several years ago, there remains an urgent need to proactively mitigate against ethics dumping dynamics that replicate the same power imbalances and normative exclusions the field was meant to redress (Powles and Nissenbaum, 2018).

## Discussion

Research ethics dumping and AI ethics dumping, while both involving the unfair transfer of ethical responsibilities, occur in distinct contexts with differing mechanisms and implications. Research ethics dumping refers to the practice of exporting ethically questionable research practices from a setting with strong governance and ethical oversight to one with weaker regulations, often in developing countries. This often results in local populations bearing the brunt of risks without corresponding benefits, as researchers exploit regulatory gaps and institutional deficiencies. In this context, ethics dumping is a product of power imbalances where ethical standards are dictated by privileged actors without consideration for the local context, leading to exploitation and harm.

AI ethics dumping, on the other hand, involves the offloading of ethical responsibilities from AI developers, guideline authors, institutional implementors, and policymakers onto end-users and communities that may not have the capacity to manage the embedded normative content of AI systems. This form of dumping arises from the complex and opaque nature of AI technologies, where the ethical implications are often encoded into the algorithms and systems themselves. The lack of transparency and the centralized development of ethical guidelines disconnected from local realities exacerbate the issue, leaving users to grapple with ethical dilemmas without adequate support or resources. Unlike research ethics dumping, which typically involves the physical relocation of research practices, AI ethics dumping is more about the diffusion of ethical burdens across a digital and global landscape, where the consequences of ethical misalignments are often invisible yet profound.

The risks of AI ethics dumping pervade AI development, ethics guidelines enunciation, institutional procurement/deployment, and statutory governance mechanisms. However, scattered across AI ethics discourse are proposals and principles that could serve as guideposts for mitigating dumping dynamics, if embraced more systematically. These value-commitments, processes, and structures emphasize empowering users/host environments, committing to inclusive stakeholder participation, enabling ethical localization while pursuing governance harmonization, and establishing clear accountability attribution across the AI lifecycle.

### Empowering users and host environments

A core ethics dumping dynamic stems from the imposition of AI systems designed elsewhere through opaque processes onto local users without substantive normative negotiation or adaptation. This concentrates normative and technical mastery with developers while users must unilaterally shoulder accountability burdens for unforeseen negative impacts. Mitigating this requires a *pull* rather than *push* approach to AI deployment, where systems are co-developed or iteratively customized to meet verified needs of host environments and align with *in-situ* value systems (Shilton, 2018). The technical and organizational specifics of AI rollout must be workable within existing user constraints, dialogically incorporating feedback on potential tensions and trade-offs (Leslie, 2019). Local users and community representatives must maintain agency and decision rights over whether, how and to what degree AI should be integrated rather than having transformations imposed upon them. Responsible AI development cannot presume to dictate conditions of use but must empower recipients with meaningful refusal rights and agenda-setting influence over ethical baselines (Tinnirello, 2022). This approach shifts the locus of normative control and ensures users are not burdened with compliance obligations disconnected from their lived realities and accountabilities. Rather than ethics dumping, the emphasis should be on co-design and sustained relational alignment between developers and users, balancing expert inputs with contextually-nuanced frontline norms in an inclusive co-reasoning process along the AI lifecycle (Pacia et al., 2024).

### Inclusive stakeholder engagement

The EU-funded TRUST project developed a code of conduct and two other tools that encourage freedom from ethics dumping in international research (Andanda et al., 2014). Such tools encourage active collaboration both internally to the EU and with countries outside the EU (Andanda et al., 2014) as suggested by the Directorate-General for Research and Innovation (2010). A similar approach in the context of AI ethics dumping would be to institutionalize inclusive stakeholder engagement processes across all AI norm-setting activities—ethics guidelines development, institutional procurement protocols, impact assessments and statutory governance frameworks. Currently, key voices are too often sidelined or marginalized in agenda-setting over AI ethics, from minority representatives and civil society advocates to frontline professionals and impacted community members (Bélisle-Pipon et al., 2022). Exclusion facilitates dumping by concentrating normative and technical control with a narrow set of actors furthest removed from downstream burdens of AI deployment. Inclusive processes must solicit, incorporate, and empower diverse stakeholder perspectives throughout each stage of the AI lifecycle—from initial problem formulation to ongoing sociotechnical audits. (Dobbe et al., 2021; Zhang et al., 2021; Riezebos et al., 2022; Lam et al., 2023).

### Enhancing accountability mechanisms in AI ethics

Robust accountability frameworks are critical to ensure that ethical standards are met in AI development and deployment (Wirtz, 2022). This necessitates the establishment of independent oversight bodies with the authority to enforce sanctions and conduct investigations into breaches of ethics (Schmitt, 2022). Transparency is paramount in these processes to enable public oversight and to offer redress for those impacted by AI technologies (Bélisle-Pipon et al., 2022). Beyond procedural measures, instilling a culture of ethics vigilance within the AI development community is essential. Such a culture encourages ongoing self-reflection, adherence to ethical principles, and the courage to see them materialize for stakeholders and end-users throughout the AI lifecycle. Through these mechanisms, it becomes possible to hold AI ethics dumpers accountable, thereby ensuring that AI technologies are developed and deployed in alignment with human rights and democratic values.

### Challenges

While the concept of ethics dumping serves as a valuable lens for examining power imbalances and accountability gaps within the AI ethics ecosystem, it is crucial to recognize the complexity involved in the distribution of ethical responsibility. In some contexts, the transfer of ethical responsibility may not only be unavoidable but also necessary. However, this necessary transfer raises a critical concern: what happens when the entities that *should* assume this responsibility lack the power or capacity to act ethically? In such situations, the transfer could result in a form of ethics dumping on certain stakeholders that, despite their intentions, may be ill-equipped to manage the ethical implications effectively. This highlights a challenge in the discourse around ethics dumping—differentiating between justified transfers of responsibility and those that unfairly burden entities incapable of upholding ethical standards. Thus, while it is important to advocate for the democratization of normative control (for instance through stakeholder engagement) and to guard against ethics dumping, it is equally important to acknowledge that not all transfers of ethical responsibility are inherently negative. Some are essential for operationalizing ethical principles. The key challenge is ensuring that these transfers are accompanied by adequate support and resources, enabling the responsible parties to fulfill their ethical obligations without perpetuating power imbalances or ethical shortcomings.

## Conclusion

The concept of ethics dumping provides a revealing lens for examining power asymmetries and accountability deficits within the AI ethics ecosystem. As this analysis has shown, practices across the AI lifecycle—from developer choices embedding normative assumptions into systems, to institutional deployments imposing transformative technologies without assessing impacts on users, to governance frameworks dictating compliance obligations while marginalizing key stakeholder voices—all risk facilitating the dumping of ethical burdens onto those least equipped to negotiate or uphold externally-imposed norms.

Enabled by AI’s opacity, autonomy, and transformative impacts, ethics dumping represents an insidious dynamic wherein the very actors most influential in shaping AI’s normative terrain are able to distance themselves from accountability for its local manifestations and ripple effects. Those “on the ground” interfacing with AI systems daily must then unilaterally shoulder responsibility for addressing ethical quandaries and negative externalities they had little part in defining or governing from the outset. If left unaddressed, ethics dumping threatens to replicate many of the same injustices, exclusions, and power imbalances the AI ethics movement overtly sought to rectify. Virtuous rhetoric around human-centered AI risks ringing hollow if it prioritizes ethics-washing over inclusive participatory mechanisms. Centralized governance aiming for harmonized norms is liable to perpetuate digital coloniality if undertaken without sustained dialogue and norm-negotiation with peripheralized stakeholders. Even the most well-intentioned ethical principles can enable ethics dumping if stakeholder representation was lacking during their conception and formalization.

Mitigating ethics dumping therefore requires multifaceted interventions that democratize normative control over AI systems while clearly delineating responsibility across the entire ecosystem. Empowering users and host environments with agency over AI deployment decisions through *pull* models and continuous feedback loops is crucial, as is fortifying stakeholder inclusion throughout all norm-setting processes. Creating space for ethical localization and on-the-ground improvisation within harmonized governance frameworks is equally vital for avoiding one-size-fits-all imposition of exogenous norms. Ultimately, a commitment to leveling asymmetries and centering those facing the brunt of AI’s impacts must be the lodestar for AI ethics going forward. Only by proactively restructuring the distribution of normative control and accountability attribution can the existential risks of ethics dumping be averted. Responsible AI innovation requires democratizing agenda-setting for an ethics of technologies in a pluralistic manner attuned to contextual realities, local priorities, and indigenous perspectives. Broad principles and centralized policies have their place, but not at the cost of perpetuating ethics dumping that undermines AI’s emancipatory potential through new modes of disenfranchisement and dispossession.

## Data Availability

The original contributions presented in the study are included in the article/supplementary material, further inquiries can be directed to the corresponding author.
